# Spectral Analysis on Time-Course Expression Data: Detecting Periodic Genes Using a Real-Valued Iterative Adaptive Approach

**DOI:** 10.1155/2013/171530

**Published:** 2013-02-28

**Authors:** Kwadwo S. Agyepong, Fang-Han Hsu, Edward R. Dougherty, Erchin Serpedin

**Affiliations:** ^1^Department of Electrical and Computer Engineering, Texas A&M University, College Station, TX 77843-3128, USA; ^2^Computational Biology Division, Translational Genomics Research Institute, Phoenix, AZ 85004-2101, USA

## Abstract

Time-course expression profiles and methods for spectrum analysis have been applied for detecting transcriptional periodicities, which are valuable patterns to unravel genes associated with cell cycle and circadian rhythm regulation. However, most of the proposed methods suffer from restrictions and large false positives to a certain extent. Additionally, in some experiments, arbitrarily irregular sampling times as well as the presence of high noise and small sample sizes make accurate detection a challenging task. A novel scheme for detecting periodicities in time-course expression data is proposed, in which a real-valued iterative adaptive approach (RIAA), originally proposed for signal processing, is applied for periodogram estimation. The inferred spectrum is then analyzed using Fisher's hypothesis test. With a proper *p*-value threshold, periodic genes can be detected. A periodic signal, two nonperiodic signals, and four sampling strategies were considered in the simulations, including both bursts and drops. In addition, two yeast real datasets were applied for validation. The simulations and real data analysis reveal that RIAA can perform competitively with the existing algorithms. The advantage of RIAA is manifested when the expression data are highly irregularly sampled, and when the number of cycles covered by the sampling time points is very reduced.

## 1. Introduction

Patterns of periodic gene expression have been found to be associated with essential biological processes such as cell cycle and circadian rhythm [1], and the detection of periodic genes is crucial to advance our understanding of gene function, disease pathways, and, ultimately, therapeutic solutions. Using high-throughput technologies such as microarrays, gene expression profiles at discrete time points can be derived and hundreds of cell cycle regulated genes have been reported in a variety of species. For example, Spellman et al. applied cell synchronization methods and conducted time-course gene expression experiments on *Saccharomyces cerevisiae* [2]. The authors identified 800 cell cycle regulated genes using DNA microarrays. Also, Rustici et al. and Menges et al. identified 407 and about 500 cell cycle regulated genes in *Schizosaccharomyces pombe* and *Arabidopsis*, respectively [3, 4].

Signal processing in the frequency domain simplifies the analysis and an emerging number of studies have demonstrated the power of spectrum analysis in the detection of periodic genes. Considering the common issues of missing values and noise in microarray experiments, Ahdesmäki et al. proposed a robust detection method incorporating the fast Fourier transform (FFT) with a series of data preprocessing and hypothesis testing steps [5]. Two years later, the authors further proposed a modified version for expression data with unevenly spaced time intervals [6]. A Lomb-Scargle (LS) approach, originally used for finding periodicities in astrophysics, was developed for expression data with uneven sampling [7]. Yang et al. further improved the performance using a detrended fluctuation analysis [8]. It used harmonic regression in the time domain for significance evaluation. The method was termed “Lomb-Scargle periodogram and harmonic regression (LSPR).” Basically, these methods consists of two steps: transferring the signals into the frequency (spectral) domain and then applying a significance evaluation test for the resulting peak in the spectral density.

While numerous methods have been developed for detecting periodicities in gene expression, most of these methods suffer from false positive errors and working restrictions to a certain extent, particularly when the time-course data contain limited time points. In addition, no algorithm seems available to resolve all of these challenges. Microarray as well as other high-throughput experiments, due to high manufacturing and preparation costs, have common characteristics of small sample size [9], noisy measurements [10], and arbitrary sampling strategies [11], thereby making the detection of periodicities highly challenging. Since the number and functions of cell cycle regulated genes, or periodic genes, remain greatly uncertain, advances in detection algorithms are urgently needed.

Recently, Stoica et al. developed a novel nonparametric method, termed the “real-valued iterative adaptive approach (RIAA),” specifically for spectral analysis with nonuniformly sampled data [12]. As stated by the authors, RIAA, an iteratively weighted least-squares periodogram, can provide robust spectral estimates and is most suitable for sinusoidal signals. These characteristics of RIAA inspired us to apply it to time-course gene expression data and conduct an examination on its performance. Herein, we incorporate RIAA with a Fisher's statistic to detect transcriptional periodicities. A rigorous comparison of RIAA with several aforementioned algorithms in terms of sensitivities and specificities is conducted through simulations and simulation results dealing with real data analysis are also provided.

In this study, we found that the RIAA algorithm can provide robust spectral estimates for the detection of periodic genes regardless of the sampling strategies adopted in the experiments or the nonperiodic nature of noise present in the measurement process. We show through simulations that the RIAA can outperform the existing algorithms particularly when the data are highly irregularly sampled, and when the number of cycles covered by the sampling time points is very few. These characteristics of RIAA fit perfectly the needs of time-course gene expression data analysis. This paper is organized as follows. In Section 2, we begin with an overview of RIAA. In Section 3, a scheme for detecting periodicities is proposed, and simulation models for performance evaluation and a real data analysis for validation purposes are presented. A complete investigation of the performance of RIAA and a rigorous comparison with other algorithms are provided in Section 4.

## 2. RIAA Algorithm

RIAA is an iterative algorithm developed for finding the least-squares periodogram with the utilization of a weighted function. The essential mathematics involved in RIAA is introduced in this section with the algorithm input being time-course expression data; for more details regarding RIAA, the readers are encouraged to check the original paper by Stoica et al. [12].

### 2.1. Basics

Suppose that the signals associated with the periodic gene expressions are composed of noise and sinusoidal components. Let *y*_*h*_(*t*_*i*_), *i* = 1,…, *n*, denote the time-course expression ratios of gene *h* at instances *t*_1_,…, *t*_*n*_, respectively; *y*_*h*_(*t*_*i*_) are real numbers; ∑_*i*=1_^*n*^ *y*_*h*_(*t*_*i*_) = 0. The least-squares periodogram Φ_*lsp*_ is given by

(1) $$\begin{array}{c} \Phi_{lsp}={\left|\hat{\alpha }(\omega )\right|}^{2}, \end{array}$$

where $\hat{\alpha }(\omega )$ is the solution to the following fitting problem:

(2) $$\begin{array}{c} \hat{\alpha }\left(\omega \right)=\arg\underset{\alpha \left(\omega \right)}{\min}\sum_{i=1}^{n}{\left[y_{h}\left(t_{i}\right)-\alpha \left(\omega \right)e^{j\omega t_{i}}\right]}^{2}. \end{array}$$

Let *α*(*ω*) = |*α*(*ω*) | *e* ^*jϕ*(*ω*)^ = *βe* ^*jθ*^, where *β* = |*α*(*ω*)|≥0 and *θ* = *ϕ*(*ω*) ∈ [0,2*π*] refer to the amplitude and phase of *α*(*ω*), respectively. The criterion in (2) can then be rewritten as

(3) $$\begin{array}{c} \sum_{i=1}^{n}{\left[y_{h}\left(t_{i}\right)-\beta \cos\left(\omega t_{i}+\theta \right)\right]}^{2}+\beta^{2}\sum_{i=1}^{n}\sin^{2}\left(\omega t_{i}+\theta \right). \end{array}$$

The second term in the above equation is data independent and can be omitted from the minimization operation. Hence, the criterion (2) is simplified to

(4) $$\begin{array}{c} \left(\hat{\beta },\hat{\theta }\right)=\arg\underset{\beta ,\theta }{\min}\sum_{i=1}^{n}{\left[y_{h}\left(t_{i}\right)-\beta \cos\left(\omega t_{i}+\theta \right)\right]}^{2}. \end{array}$$

We further apply *a* = *β*cos⁡(*θ*) and *b* = −*β*sin(*θ*) and derive an equivalent of (4) as follows:

(5) $$\begin{array}{c} \left(\hat{a},\hat{b}\right)=\arg\underset{a,b}{\min}\sum_{i=1}^{n}{\left[y_{h}\left(t_{i}\right)-a\cos\left(\omega t_{i}\right)-b\sin\left(\omega t_{i}\right)\right]}^{2}. \end{array}$$

The target of interest to the fitting problem now becomes $\hat{a}$ and $\hat{b}$ (instead of *α*(*ω*)), and the solution is well known to be

(6) $$\begin{array}{c} \left[\begin{array}{c} \hat{a}\\ \hat{b} \end{array}\right]=R^{-1}r, \end{array}$$

where

(7) $$\begin{array}{c} R=\sum_{i=1}^{n}\left[\begin{array}{cc} \cos{\left(\omega t_{i}\right)}^{2} & \cos\left(\omega t_{i}\right)\sin\left(\omega t_{i}\right)\\ \sin\left(\omega t_{i}\right)\cos\left(\omega t_{i}\right) & \sin{\left(\omega t_{i}\right)}^{2} \end{array}\right],\\ r=\sum_{i=1}^{n}\left[\begin{array}{c} \cos\left(\omega t_{i}\right)\\ \sin\left(\omega t_{i}\right) \end{array}\right]y_{h}\left(t_{i}\right). \end{array}$$

After $\hat{a}$ and $\hat{b}$ are estimated, the least-squares periodogram can be derived.

### 2.2. Observation Interval and Resolution

Prior to implementation of RIAA for periodogram estimation, the observation interval [0, *ω*_max⁡_] and the resolution in terms of grid size have to be selected. To this end, the maximum frequency *ω*_max⁡_ in the observation interval without aliasing errors for sampling instances *t*_1_,…, *t*_*n*_, can be evaluated by

(8) $$\begin{array}{c} \omega_{\max}=\frac{\omega_{0}}{2}, \end{array}$$

where *ω*_0_ is given by

(9) $$\begin{array}{c} \omega_{0}=\frac{2\left(n-1\right)\pi }{\sum_{i=1}^{n-1}\left(t_{i+1}-t_{i}\right)}. \end{array}$$

The observation interval [0, *ω*_max⁡_] is hence chosen after *ω*_max⁡_ is obtained.

To ensure that the smallest frequency separation in time-course expression data with regular or irregular sampling can be adequately detected, the grid size Δ*ω* is chosen to be

(10) $$\begin{array}{c} \Delta \omega =\frac{2\pi }{t_{n}-t_{1}}, \end{array}$$

which, in fact, is the resolution limit of the least-squares periodogram. As a result, the frequency grids *ω*_*g*_ considered in periodogram are

(11) $$\begin{array}{c} \omega_{g}=g\Delta \omega ,\;g=1,\ldots ,G, \end{array}$$

where the number of grids *G* is given by

(12) $$\begin{array}{c} G=\left\lfloor \frac{\omega_{\max}}{\Delta \omega }\right\rfloor . \end{array}$$

### 2.3. Implementation

The following notations are introduced for the implementation of RIAA at a specific frequency *ω*_*g*_:

(13) $$\begin{array}{c} Y={\left[\begin{array}{c} y_{h}\left(t_{1}\right)\;\;\;\cdots \;\;\;\;y_{h}\left(t_{n}\right) \end{array}\right]}^{T},\\ \rho_{g}={\left[\begin{array}{c} a\left(\omega_{g}\right)\;\;\;\;b\left(\omega_{g}\right) \end{array}\right]}^{T},\\ A_{g}=\left[\begin{array}{c} c_{g}\;\;\;\;s_{g} \end{array}\right], \end{array}$$

where

(14) $$\begin{array}{c} c_{g}={\left[\begin{array}{c} \cos\left(\omega_{g}t_{1}\right)\;\;\;\cdots \;\;\;\;\cos\left(\omega_{g}t_{n}\right) \end{array}\right]}^{T},\\ s_{g}={\left[\begin{array}{c} \sin\left(\omega_{g}t_{1}\right)\;\;\;\cdots \;\;\;\;\sin\left(\omega_{g}t_{n}\right) \end{array}\right]}^{T}, \end{array}$$

and *a*(*ω*_*g*_) and *b*(*ω*_*g*_) denote variables *a* and *b* at frequency *ω*_*g*_, respectively.

RIAA's salient feature is the addition of a weighted matrix **Q**_*g*_ to the least-squares fitting criterion. The weighted matrix **Q**_*g*_ can be viewed as a covariance matrix encapsulating the contributions of noise and other sinusoidal components in **Y** other than *ω*_*g*_ to the spectrum; it is defined as

(15) $$\begin{array}{c} Q_{g}=\Sigma +\sum_{m=1,m\neq g}^{G}A_{m}D_{m}A_{m}^{T}, \end{array}$$

where

(16) $$\begin{array}{c} D_{m}=\frac{a^{2}\left(\omega_{g}\right)+b^{2}\left(\omega_{g}\right)}{2}\left[\begin{array}{c} 1\;\;\;\;0\\ 0\;\;\;\;1 \end{array}\right], \end{array}$$

and Σ denotes the covariance matrix of noise in expression data **Y**, given by

(17) $$\begin{array}{c} \Sigma =\left[\begin{array}{c} \sigma^{2}\;\;\;\;\ldots \;\;\;\;0\\ \vdots \;\;\;\;\ddots \;\;\;\;\vdots \\ 0\;\;\;\;\ldots \;\;\;\;\sigma^{2} \end{array}\right]. \end{array}$$

Assuming that **Q**_*g*_ is invertible, in RIAA, a weighted least-squares fitting problem is formulated and considered for finding $\hat{a}$ and $\hat{b}$ (instead of using (5)), and it is written in the form of matrices using (13) as follows:

(18) $$\begin{array}{c} {\hat{\rho }}_{g}=\arg\underset{\rho_{g}}{\min}{\left[Y-A_{g}\rho_{g}\right]}^{T}Q_{g}^{-1}\left[Y-A_{g}\rho_{g}\right]. \end{array}$$

In Stoica et al. [12], the solution to (18) has been shown to be

(19) $$\begin{array}{c} {\hat{\rho }}_{g}=\frac{A_{g}^{T}Q_{g}^{-1}Y}{A_{g}^{T}Q_{g}^{-1}A_{g}}, \end{array}$$

and the RIAA periodogram at *ω* = *ω*_*g*_ can be derived by

(20) $$\begin{array}{c} \Phi_{\text{riaa}}\left(\omega_{g}\right)=\frac{1}{n}{\hat{\rho }}_{g}^{T}\left(A_{g}^{T}A_{g}\right){\hat{\rho }}_{g}. \end{array}$$

From (15) and (19), it is obvious that **Q**_*g*_ and ${\hat{\rho }}_{g}$ are dependent on each other. An iterative approach (i.e., RIAA) is hence a feasible solution to get the estimate ${\hat{\rho }}_{g}$ and the weighted matrix **Q**_*g*_.

The iteration for estimating spectrum starts with initial estimates ${\hat{\rho }}_{g}^{0}$, in which the elements $\hat{a}$ and $\hat{b}$ are given by (6) with *ω* = *ω*_*g*_, *g* = 1,…, *G*. After initialization, the first iteration begins. First, the elements $\hat{a}$ and $\hat{b}$ of ${\hat{\rho }}_{g}^{0}$ are applied to obtain ${\hat{D}}_{m}^{1}$ using (16). Secondly, to get a good estimate of ${\hat{\sigma }}^{1}$, the frequency *ω*_*p*_ at which the largest value-*p* is located in the temporary periodogram Φ^0^(*ω*_*g*_), *g* = 1,…, *G*, derived using (20) with ${\hat{\rho }}_{g}={\hat{\rho }}_{g}^{0}$, is applied for obtaining a reversed engineered signal ${\hat{Y}}^{0}$. The elements ${\hat{y}}_{h}\left(t_{i}\right),\;i=1,\ldots ,n$, in ${\hat{Y}}^{0}$ are given by

(21) $$\begin{array}{c} {\hat{y}}_{h}\left(t_{i}\right)=\sqrt{2P}\cos\left(\omega_{p}t_{i}+s\right),\;i=1,\ldots ,n. \end{array}$$

The phase of the cosine function *s* is unknown; however, ${\hat{\sigma }}^{1}$ is estimable using

(22) $$\begin{array}{c} {\hat{\sigma }}^{1}=\underset{s\in \left[0,2\pi \right]}{\min}\frac{{||Y-{\hat{Y}}^{0}||}^{2}}{n}, \end{array}$$

where ||·|| is the Euclidean norm. With estimates ${\hat{D}}_{m}^{1}$ and ${\hat{\sigma }}^{1}$, the estimates ${\hat{Q}}_{g}^{1}$, *g* = 1,…, *G*, in the first iteration are hence given by (15). After this, ${\hat{Q}}_{g}^{1}$ are inserted into the right-hand side of (19) and updated estimates ${\hat{\rho }}_{g}^{1}$, *g* = 1,…, *G*, are derived. The algorithm consists of repeating these steps and updating ${\hat{Q}}_{g}^{k}$ and ${\hat{\rho }}_{g}^{k}$ iteratively, where *k* denotes the number of iterations, until a termination criterion is reached. If the process stops at the *K*th iteration, then the final RIAA periodogram is given by (20) using ${\hat{\rho }}_{g}^{K}$. The pseudocode in Algorithm 1 represents a concise description of the iterative RIAA process.

## 3. Methods

**Figure 1:**
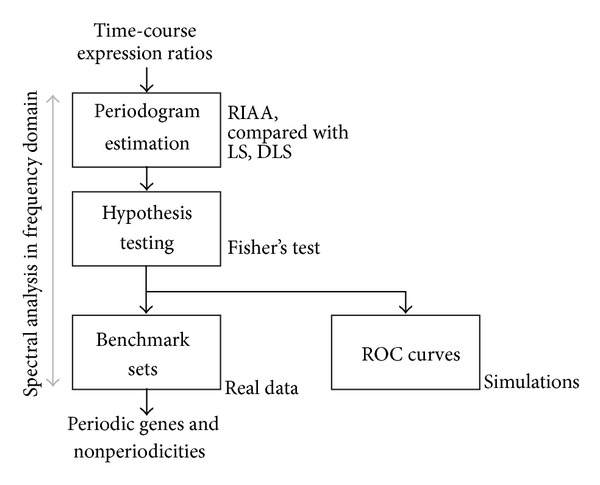
The scheme of the process for detecting periodicities in time-course expression data.

Figure 1 demonstrates our scheme for periodicity detection and algorithm comparison. The first step involves a periodogram estimation, which converts the time-course gene expression ratios into the frequency domain. Three methods are considered for comparison: RIAA, LS, and a detrend LS (termed DLS), which uses an additional detrend function (developed in LSPR) before regular LS periodogram estimation is applied. The derived spectra are then analyzed using hypothesis testing. This study is conducted using a Fisher's test, with the null hypothesis that there are no periodic signals in the time domain and hence no significantly large peak in the derived spectra. The algorithm performance is evaluated and compared via simulations and receiver operating characteristic (ROC) curves. In real microarray data analysis, three published benchmark sets are utilized as standards of cell cycle genes for performance comparison.

### 3.1. Fisher's Test

After the spectrum of time-course expression data is obtained via periodogram estimation, a Fisher's statistic *f* for gene *h* with the null hypothesis *H*_0_ that the peak of the spectral density is insignificant against the alternative hypothesis *H*_1_ that the peak of the spectral density is significant is applied as

(23) $$\begin{array}{c} f_{h}=\frac{\max_{1\leq g\leq G}\left(\Phi \left(\omega_{g}\right)\right)}{G^{-1}\sum_{g=1}^{G}\Phi \left(\omega_{g}\right)}, \end{array}$$

where Φ refers to the periodogram derived using RIAA, LS, or DLS. The null hypothesis *H*_0_ is rejected, and the gene *h* is claimed as a periodic gene if its *p*-value, denoted as *p*_*h*_, is less than or equal to a specific significance threshold. For simplicity, *p*_*h*_ is approximated from the asymptotic null distribution of *f* assuming Gaussian noise [13] as follows:

(24) $$\begin{array}{c} p_{h}=1-e^{-ne^{-f_{h}}}. \end{array}$$

In real data analysis, deviation might be invoked for the estimation of *p*_*h*_ when the time-course data is short. This issue was carefully addressed by Liew et al. [14], and, as suggested, alternative methods such as random permutation may provide less deviation and better performance. However, permutation also has limitations such as tending to be conservative [15]. While finding the most robust method for the *p*-value evaluation remains an open question, it gets beyond the scope of this study since the algorithm comparison via ROC curves is threshold independent [16], and the results are unaffected by the deviation.

### 3.2. Simulations

Simulations are applied to evaluate the performance of RIAA. The simulation models and sampling strategies used for simulations are described in the following paragraphs.

#### 3.2.1. Periodic and Nonperiodic Signals

Three models, one for periodic signals and two for nonperiodic signals, are considered as transcriptional signals. Since periodic genes are transcribed in an oscillatory manner, the expression levels *y*_*s*_ embedded with periodicities are assumed to be

(25) $$\begin{array}{c} y_{s}\left(t_{i}\right)=M\cos\left(\omega_{s}t_{i}\right)+\epsilon_{t_{i}},\;i=1,\ldots ,n, \end{array}$$

where *M* denotes the sinusoidal amplitude; *ω*_*s*_ refers to the signal frequency; *ϵ*_*t*_*i*__ are Gaussian noise independent and identically distributed (i.i.d.) with parameters *μ* and *σ*. For nonperiodic signals, the first model *y*_*n*_ is simply composed of Gaussian noise, given by

(26) $$\begin{array}{c} y_{n}\left(t_{i}\right)=\epsilon_{t_{i}},\;i=1,\ldots ,n. \end{array}$$

Additionally, as visualized by Chubb et al., gene transcription can be nonperiodically activated with irregular intervals in a living eukaryotic cell, like pulses turning on and off rapidly and discontinuously [17]. Based on this, the second nonperiodic model *y*_*n*_′ incorporates one additional transcriptional burst and one additional sudden drop into the Gaussian noise, which can be written as

(27) $$\begin{array}{c} y_{n}^{\prime }\left(t_{i}\right)=I_{b}\left(t_{i}\right)-I_{d}\left(t_{i}\right)+\epsilon_{t_{i}},\;i=1,\ldots ,n, \end{array}$$

where *I*_*b*_ and *I*_*d*_ are indicator functions, equal to 1 at the location of the burst and the drop, respectively, and 0 otherwise. The transcriptional burst assumes a positive pulse while the transcriptional drop assumes a negative pulse. Both of them may be located randomly among all time points and are assumed to last for two time points. In other words, the indicator functions are equal to 1 at two consecutive time points, say, *I*_*b*_ = 1 at *t*_*i*_ and *t*_*i*+1_. The burst and the drop have no overlap.

#### 3.2.2. Sampling Strategies

As for the choices of sampling time points *t*_*i*_, *i* = 1,…, *n*, four different sampling strategies, one with regular sampling and three with irregular sampling, are considered. First, regular sampling is applied in which all time intervals are set to be 1/*c*, where *c* is a constant. Secondly, a bio-like sampling strategy is invoked. This strategy tends to have more time points at the beginning of time-course experiments and less time points after we set the first 2/3 time intervals as 1/*c* and set the next 1/3 time intervals as 2/*c*. Third, time intervals are randomly chosen between 1/*c* and 2/*c*. The last sampling strategy, in which all time intervals are exponentially distributed with parameter *c*, is less realistic than the others but it is helpful for us to evaluate the performance of RIAA under pathological conditions.

ROC curves are applied for performance comparison. To this end, 10,000 periodic signals were generated using (25) and 10,000 nonperiodic signals were generated using either (26) or (27). Sensitivity measures the proportion of successful detection among the 10,000 periodic signals and specificity measures the proportion of correct claims on the 10,000 nonperiodic simulation datasets. Sampling time points are decided by one of the four sampling strategies and the number of time points *n* is chosen arbitrarily. For all ROC curves in Section 4, *c* = 2 and *n* = 24.

### 3.3. Real Data Analysis

Two yeast cell cycle experiments synchronized using an alpha-factor, one conducted by Spellman et al. [2] and one conducted by Pramila et al. [18], are considered for a real data analysis. The first time-course microarray data, termed dataset alpha and downloaded from the Yeast Cell Cycle Analysis Project website (http://genome-www.stanford.edu/cellcycle/), harbors 6,178 gene expression levels and 18 sampling time points with a 7-minute interval. The second time-course data, termed dataset alpha 38, is downloaded from the online portal for Fred Hutchinson Cancer Research Center's scientific laboratories (http://labs.fhcrc.org/breeden/cellcycle/). This dataset contains 4,774 gene expression levels and 25 sampling time points with a 5-minute interval. Three benchmark sets of genes that have been utilized in Lichtenberg et al. [19] and Liew et al. [20] as standards of cell cycle genes are also applied herein for performance comparison. These benchmark sets, involving 113, 352, and 518 genes, respectively, include candidates of cycle cell regulated genes in yeast proposed by Spellman et al. [2], Johansson et al. [21], Simon et al. [22], Lee et al. [23], and Mewes et al. [24] and are accessible in a laboratory website (http://www.cbs.dtu.dk/cellcycle/).

## 4. Results

**Figure 2:**
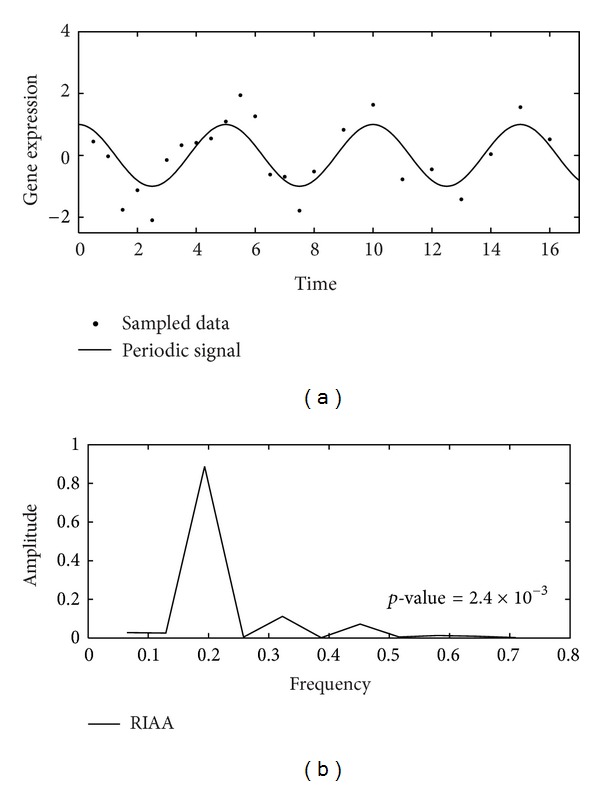
(a) A time-course periodic signal with frequency = 0.2 sampled by the bio-like sampling strategy; 16 time points are assigned to the interval (0,8], and 8 time points are assigned to the interval (8,16]. (b) The periodogram derived using RIAA. The maximum value (peak) in the periodogram locates at frequency = 0.195.

RIAA performed well in the conducted simulations. As shown in Figure 2(a), a periodic signal (solid line) with amplitude *M* = 1 and frequency *ω*_*s*_ = 0.4*π*  is sampled using the bio-like sampling strategy, which applies 16 time points in (0,8] and 8 more time points in (8,16]. Gaussian noise with parameters *μ* = 0 and *σ* = 0.5 is assumed during microarray experiments. The resulting time-course expression levels (dots), at a total of 24 time points and the sampling time information were treated as inputs to the RIAA algorithm.  Figure 2(b) demonstrates the result of periodogram estimation. In this example, the grid size Δ*ω* was chosen to be 0.065 and a total of 11 amplitudes corresponding to different frequencies were obtained and shown in the spectrum. Using Fisher's test, the peak at the third grid (frequency = 0.195) was found to be significantly large (*p*-value = 2.4 × 10  ^−3^), and hence a periodic gene was claimed.

**Figure 3:**
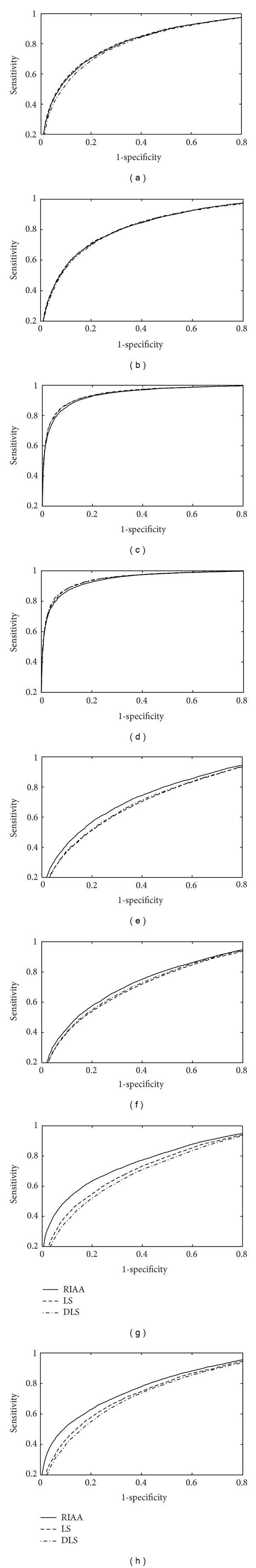
The ROC curves derived from simulations with 24 sampling time points, signal amplitude *M* = 1, *ω*_*s*_ = 0.4*π*, and Gaussian noise *μ* = 0 and *σ* = 0.5. Description of subplots is provided in Section 4.

**Figure 4:**
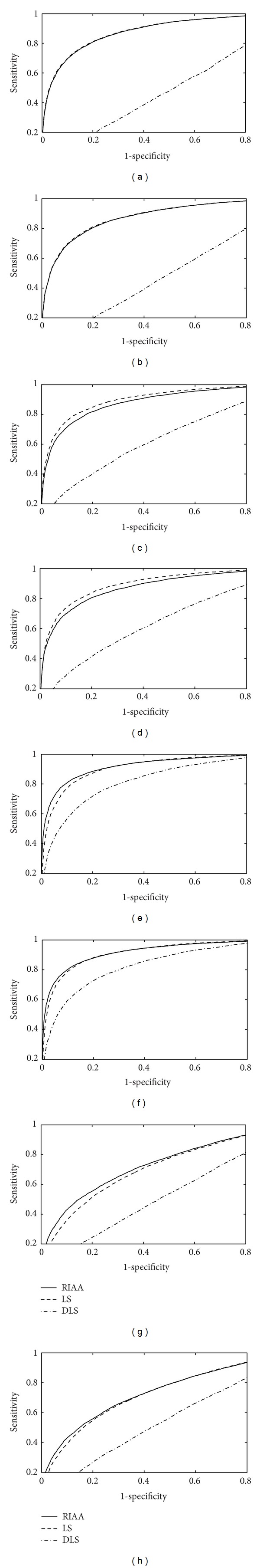
The ROC Curves derived from simulations with 24 sampling time points, signal amplitude *M* = 1, *ω*_*s*_ = 0.1*π*, and Gaussian noise *μ* = 0 and *σ* = 0.5. Description of subplots is provided in Section 4.

ROC curves strongly illustrate the performance of RIAA. In Figures 3 and 4, subplots (a)-(b), (c)-(d), (e)-(f), and (g)-(h) refer to the simulations with regular, bio-like, binomially random, and exponentially random sampling strategies, respectively. Additionally, in the left-hand side subplots (a), (c), (e), and (g), nonperiodic signals were simply Gaussian noise with parameters *μ* = 0 and *σ* = 0.5, while in the right-hand side subplots (b), (d), (f), and (h), nonperiodic signals involve not only the Gaussian noise but also a transcriptional burst and a sudden drop (27). Periodic signals were generated using (25) with amplitude *M* = 1, *c* = 2, and *n* = 24. The only difference in simulation settings between Figures 3 and 4 is the frequency of periodic signals; they are *ω*_*s*_ = 0.4*π* and 0.1*π*, respectively. As shown in these figures, LS and DLS can perform well as RIAA when the time-course data are regularly sampled, or mildly irregularly sampled; however, when data are highly irregularly sampled, RIAA outperforms the others. The superiority of RIAA over DLS is particularly clear when the signal frequency is small.

**Figure 5:**
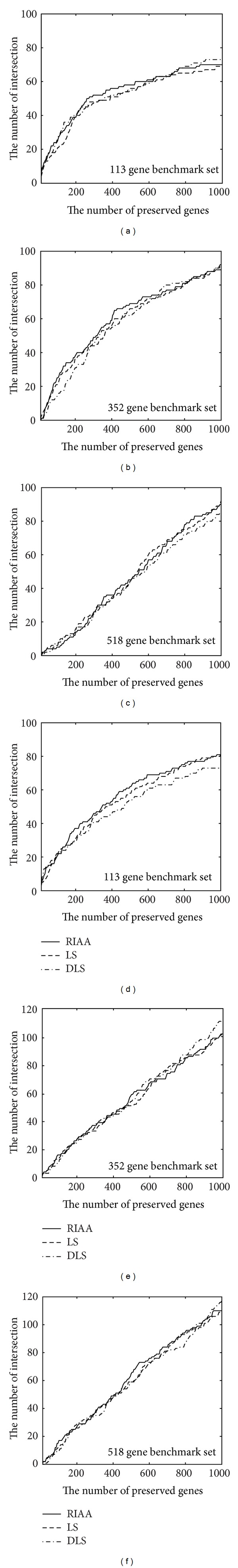
The intersection of preserved genes and the benchmark sets using RIAA, LS, and DLS algorithms. (a), (b), and (c) reveal the analysis results when dataset alpha was applied. (d), (e), and (f) reveal the analysis results when dataset alpha 38 was applied.

Figure 5 illustrates the results of the real data analysis when these three algorithms, namely, the RIAA, LS, and DLS, were applied. On the *x*-axis, the numbers indicate the thresholds *η* that we preserved and classified as periodicities among all yeast genes; on the y-axis, the numbers refer to the intersection of *η* preserved genes and the proposed periodic candidates listed in the benchmark sets. Figures 5(a)–5(c) demonstrate the results derived from dataset alpha when the 113-gene benchmark set, 352-gene benchmark set, and 518-gene benchmark set were applied, respectively. Similarly, Figures 5(d)–5(f) demonstrate the results derived from dataset alpha 38. The RIAA does not result in significant differences in the numbers of intersections when compared to those corresponding to LS and DLS in most of these cases. However, RIAA shows slightly better coverage when the dataset alpha 38 and the 113-gene benchmark set was utilized (Figure 5(d)).

## 5. Conclusions

In this study, the rigorous simulations specifically designed to comfort with real experiments reveal that the RIAA can outperform the classical LS and modified DLS algorithms when the sampling time points are highly irregular, and when the number of cycles covered by sampling times is very limited. These characteristics, as also claimed in the original study by Stoica et al. [12], suggest that the RIAA can be generally applied to detect periodicities in time-course gene expression data with good potential to yield better results. A supplementary simulation further shows the superiority of RIAA over LS and DLS when multiple periodic signals are considered (see Supplementary Figure  s1 available online at http://dx.doi.org/10.1155/2013/171530). From the simulations, we also learned that the addition of a transcriptional burst and a sudden drop to nonperiodic signals (the negatives) does not affect the power of RIAA in terms of periodicity detection. Moreover, the detrend function in DLS, designed to improve LS by removing the linearity in time-course data, may fail to provide improved accuracy and makes the algorithm unable to detect periodicities when transcription oscillates with a very low frequency.

The intersection of detected candidates and proposed periodic genes in the real data analysis (Figure 5) does not reveal much differences among RIAA, LS, and DLS. One possible reason is that the sampling time points conducted in the yeast experiment are not highly irregular (not many missing values are included), since, as demonstrated in Figures 3(a)–3(d), the RIAA just performs equally well as the LS and DLS algorithms when the time-course data are regularly or mildly irregularly sampled. Also, the very limited time points contained in the dataset may deviate the estimation of *p*-values [14] and thus hinder the RIAA from exhibiting its excellence. Besides, the number of true cell cycle genes included in the benchmark sets remains uncertain. We expect that the superiority of RIAA in real data analysis would be clearer in the future when more studies and more datasets become available.

Besides the comparison of these algorithms, it is interesting to note that the bio-like sampling strategy could lead to better detection of periodicities than the regular sampling strategy (as shown in Figures 3(c) and 3(d)). It might be beneficial to apply loose sampling time intervals at posterior periods to prolong the experimental time coverage when the number of time points is limited.

## Supplementary Material

Supplementary Figure s1: demonstrates the simulation results considering multiple periodic signals. Two sinusoidal waves with different frequency settings are superimposed as the periodic signal, and RIAA, LS, and DLS are applied for comparison.

## Figures and Tables

**Algorithm 1:**
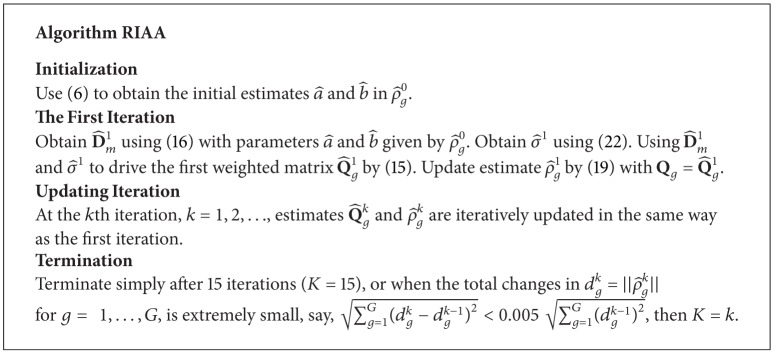
The pseudocode of the iterative process in RIAA.
