# Supplementary material for: Spectral Analysis on Time-Course Expression Data: Detecting Periodic Genes Using a Real-Valued Iterative Adaptive Approach
Source: Adv Bioinformatics. 2013 Feb 28;2013:171530. doi: 10.1155/2013/171530 (PMC3600260; doi:10.1155/2013/171530)
Supplement: Supplementary file 1 — Supplementary Figure s1: demonstrates the simulation results considering multiple periodic signals. Two sinusoidal waves with different frequency settings are superimposed as the periodic signal, and RIAA, LS, and DLS are applied for comparison. [file 171530.f1.pdf]

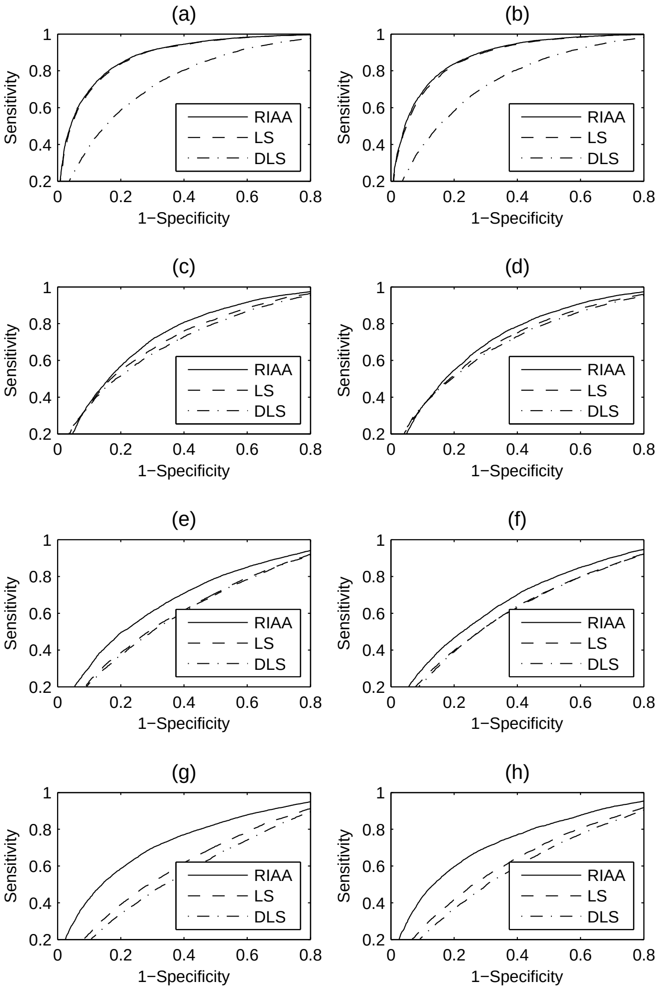

Supplementary Figure 1: The ROC Curves derived from simulations with 24 sampling time points. Periodic signals are two superimposed sinusoidal waves with amplitudes $M_1 = M_2 = 1$, frequencies $\omega_{s1} = 0.4\pi$ and $\omega_{s2} = 0.2\pi$. Sampling strategies: (a)(b) regular, (c)(d) bio-like, (e)(f) binomially random, (g)(h) exponentially random. The left-hand side sub-plots (a, c, e, and g) consider non-periodic signals with Gaussian noise $\mu = 0$ and $\sigma = 0.5$. The right-hand side sub-plots (b, d, f, and h) additionally consider a transcriptional burst and a sudden drop (Eq.(27)).
